# Enhanced MFC power production and struvite recovery by the addition of sea salts to urine

**DOI:** 10.1016/j.watres.2016.11.017

**Published:** 2017-02-01

**Authors:** Irene Merino-Jimenez, Veronica Celorrio, David J. Fermin, John Greenman, Ioannis Ieropoulos

**Affiliations:** a Bristol BioEnergy Centre, Bristol Robotics Laboratory, University of the West of England, BS16 1QY, UK; b School of Chemistry, University of Bristol, Cantock's Close, Bristol, BS8 1TS, UK; c Biological, Biomedical and Analytical Sciences, University of the West of England, BS16 1QY, UK

**Keywords:** Microbial fuel cell (MFC), Ceramic membrane, Struvite recovery, Catholyte generation, Urine treatment

## Abstract

Urine is an excellent fuel for electricity generation in Microbial Fuel Cells (MFCs), especially with practical implementations in mind. Moreover, urine has a high content in nutrients which can be easily recovered. Struvite (MgNH_4_PO_4_·6H_2_O) crystals naturally precipitate in urine, but this reaction can be enhanced by the introduction of additional magnesium. In this work, the effect of magnesium additives on the power output of the MFCs and on the catholyte generation is evaluated. Several magnesium sources including MgCl_2_, artificial sea water and a commercially available sea salts mixture for seawater preparation (SeaMix) were mixed with real fresh human urine in order to enhance struvite precipitation. The supernatant of each mixture was tested as a feedstock for the MFCs and it was evaluated in terms of power output and catholyte generation. The commercial SeaMix showed the best performance in terms of struvite precipitation, increasing the amount of struvite in the solid collected from 21% to 94%. Moreover, the SeaMix increased the maximum power performance of the MFCs by over 10% and it also changed the properties of the catholyte collected by increasing the pH, conductivity and the concentration of chloride ions. These results demonstrate that the addition of sea-salts to real urine is beneficial for both struvite recovery and electricity generation in MFCs.

## Highlights

• Electricity generation from MFCs increased by 10% adding SeaSalts to urine.
• Struvite precipitation from urine increased by adding SeaSalts.
• Struvite recovery from urine increased from 21% to 94%.
• Catholyte generated from MFCs treating urine, affected by feedstock composition.

## Graphical abstract

**Figure:**
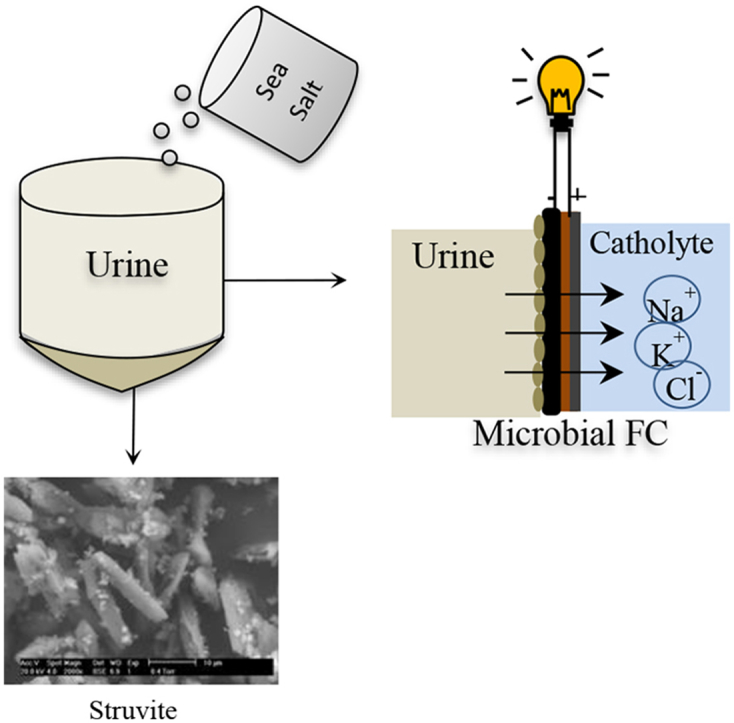

## 1 Introduction

In the past decade, struvite crystallization has gained interest as a route to phosphorus and nitrogen recovery (Doyle and Parsons, 2002, Doyle et al., 2003, Wilsenach et al., 2007). Struvite is an eco-friendly phosphate fertilizer known as magnesium ammonium phosphate hexahydrate (NH_4_MgPO_4_·6H_2_O). Its crystallization occurs in an equimolecular concentration of Mg^2+^, NH_4_^+^ and PO_4_^3−^ at slightly alkaline conditions according to the following reaction (Bouropoulos and Koutsoukos, 2000):

(1) $$Mg^{2+}+NH_{4}^{+}+H_{2}PO_{4}^{-}+6H_{2}O\to MgNH_{4}PO_{4}\cdot 6H_{2}O+2H^{+}$$

Mg^2+^, NH_4_^+^ and PO_4_^3−^, together with organic matter and different macro and micro elements can be found in wastewater streams (Rahman et al., 2014, Deng et al., 2006, Suzuki et al., 2007, Liu et al., 2011a, Liu et al., 2011b, Liu et al., 2011c). In domestic wastewater, urine contributes 50–80% of the phosphorous concentration and 75% of the nitrogen concentration is excreted in the form of urea (Rodríguez Arredondo et al., 2015, Larsen and Gujer, 1996, Diem and Lentner, 1970, Rubio-Rincón et al., 2014). Thus, urine is a potential source of struvite, as well as, an excellent fuel for electricity generation in MFCs (Ieropoulos et al., 2012). However, magnesium is the limiting ion for struvite formation due to its low concentration in urine and the addition of a magnesium source is therefore essential to maintain a favourable struvite precipitation rate (Anderson, 2008). Several studies have reported different magnesium sources, including MgSO_4_, MgO, Mg(OH)_2_ and MgCl_2_, that if added to the wastewater or urine, can increase the reaction rate of struvite precipitation. Yetilmezsoy and Zengin (2009) found no significant differences comparing MgCl_2_ and MgSO_4_; however, the most common magnesium source used for this purpose is MgCl_2_, since it is believed that higher efficiencies of NH_4_^+^-N, COD and colour removals can be achieved. In an effort to obtain a low cost phosphate recovery technique, Ye et al. (2011) reported the use of bittern, the waste brine remaining after salt (NaCl) extraction from seawater, as a magnesium source to precipitate struvite from wastewater, obtaining over 90% phosphate removal and 23–29% ammonium reduction. More recently, the use of seawater as a natural and free MgCl_2_ source to precipitate struvite from urine has also been shown to be an economical and effective technique for chemical struvite crystallization (Rubio-Rincón et al., 2014, Tang et al., 2015).

The efficiency of the struvite crystallization depends on several factors, including the concentration and molar ratios of Mg^2+^, NH_4_^+^ and PO_4_^3−^, pH levels and temperature, the aeration rate and the presence of Ca^2+^ (Stratful et al., 2001). Investigations have been carried out to obtain the most effective phosphate-to-magnesium ratio (PO_4_^3−^:Mg^2+^) for the optimum struvite precipitation. Rahman et al. (2011) obtained a crystallization of 93% of the phosphate present in animal waste-water by adding 1:1 molar ratio of Mg with respect to phosphate. The crystal growth and size can also be affected by the PO_4_:Mg molar ratio. Kozik et al. (2011) obtained an increased crystal size of 67 μm after 1 h reaction at pH 9 when the molar ratio was 1:1.2 compared to 80 μm when the molar ratio was 1:1. Therefore, the optimum PO_4_:Mg ratios are between 1:1 and 1:1.2, since larger crystals and high efficiency are obtained at those ratios (Koralewska et al., 2009, Matynia et al., 2013, Huang et al., 2012, Hutnik et al., 2013a, Hutnik et al., 2013b).

The concentration of NH_4_^+^ in urine can considerably decrease to almost half due to the urea hydrolysis which takes place in the presence of the enzyme urease (Rubio-Rincón et al., 2014, Kim et al., 2007):

(2) $${\left(NH_{2}\right)}_{2}CO+H_{2}O\overset{urease}{\to }NH_{3}+H_{2}NCOOH\to 2NH_{3\left(gas\right)}+CO_{2\left(gas\right)}$$

Rubio-Rincón et al. (2014) showed that the difference in the concentration of phosphate was 30% higher in the fresh urine than in urine after 24 h of hydrolysis. However, the precipitation rate was faster when using hydrolysed compared to not hydrolysed urine. After 10 min of mixing urine with the Mg source, 99.7% phosphate was converted to struvite when using hydrolysed urine, compared to 56.4% when using not hydrolysed fresh urine (Rubio-Rincón et al., 2014). This was attributed to an increase in the pH during the hydrolysis, affecting the precipitation rate and the quality of the crystals, which crystalized in its pure form near neutral pH. Nevertheless, higher P and N removal has been obtained at higher pH levels, between 7.5 and 9 (Hao et al., 2008). Considering that the urine pH can vary from 6, when fresh, to 9.5, after 24 h hydrolysis, the operating pH should be raised up to 8.3 to get more than 90% phosphorous removal (Rahman et al., 2014, Adnan et al., 2003). The pH also plays an important role during the ammonia stripping, where ammonium ions are transformed into gaseous ammonia. The concentration of NH_4_^+^ in urine can also be affected by aeration, since the air flow agitates the solution producing an increase in ammonia volatilization (Rahman et al., 2014). A decrease in the concentration of NH_4_^+^ will lead to a decrease in the struvite recovery through equation (1). The presence of Ca^2+^ in the wastewater or urine can also inhibit the crystallization due to the more favourable reaction of phosphates to generate calcium phosphates, hydroxyapatite, dicalcium phosphate, and octacalcium phosphate (Kim et al., 2007; Le Corre et al., 2005, Tisdale et al., 1985).

### 1.1 Struvite morphology

Pure struvite might precipitate in different forms: crystalline powder, single crystals of different sizes or gelatinous mass. Different colours can also be distinguished, including white, yellowish or brownish white (Lee et al., 2009, Munch and Barr, 2001, Rahman et al., 2014). It is known that the Magnesium Ammonium Phosphate (MAP) crystals can assume a number of morphologies including arrow-head, X-shape, coffin, wedge, short prismatic, or short tabular forms (Wierzbicki et al., 1997). Struvite crystal with similar morphology can be obtained using potassium instead of ammonia to form Magnesium Potassium Phosphate (MKP). Li et al. (2015) suggested that the biogenic X-shaped and unusual tabular struvite may represent two different growth stages, and that the aspartic acid-rich proteins, such as peptide chains in urinary proteins found in urine, can be responsible for the specific morphogenesis of biogenic struvite. The authors established a relationship between the crystal growth and the concentration of these proteins in urine, reaching lower growth rates at higher concentrations of proteins. Without peptide, struvite grows rapidly and a mass of penniform crystals with a length of ca. 190 mm can be obtained. The pH is another factor affecting the crystal growth and morphology, decreasing the size at lower pH (Prywer et al., 2012).

### 1.2 Microbial fuel cells for struvite recovery

Ichihashi and Hirooka (2012) recently used microbial fuel cells to precipitate struvite from swine wastewater. Unlike other experiments where MFCs were used to recover struvite, in this case phosphate ions precipitated on the cathode chamber, due to the more favourable alkaline conditions than those in the anolyte (pH = 7.2). At the cathode electrode, the oxygen reduction reaction (ORR), consumes water to produce hydroxide ions, causing a local increase of pH up to and above 9, creating ideal conditions for a fast struvite precipitation (Fornero et al., 2010). However, this might lead to a catalyst deactivation or blockage, decreasing the MFC performance. A decrease in power can also be observed after struvite precipitates in the anode chamber and it accumulates at the bottom of the MFCs, affecting the utilisable volume. Therefore, the precipitation of struvite prior to feeding would be a double advantage as both (i) an easy way of struvite recovery, and (ii) a mechanism for combating potential blockages. The use of MFCs connected to a urinal has already been reported showing electricity production capable of powering LED lights (Ieropoulos et al., 2016). Meanwhile, ammonium and phosphate could be recovered from the MFCs by the addition of MgCl_2_ to the urine (Kuntke et al., 2012, Tang et al., 2014, You et al., 2015), with no negative effect on the power (You et al., 2015). However, the addition of MgCl_2_ implies a relatively high cost for struvite production. If a free Mg source such as seawater was added to the urine, the struvite production cost would be considerably reduced (Rubio-Rincón et al., 2014). Besides electricity generation, MFCs have also been reported to produce catholyte (Gajda et al., 2014). The use of carbonaceous materials has been suggested as a low cost and long lasting alternative for the cathode electrodes (Santoro et al., 2013) and it has previously been used in MFCs generating catholyte from wastewater (Gajda et al., 2014). The catholyte is the electrolyte accumulated in the cathode chamber in MFCs with an anodic chamber and an open to air cathodic chamber, separated by a semipermeable membrane. A number of factors affect the catholyte generation including: (i) the reaction products of the ORR taking place in the cathode electrode; (ii) the hydrostatic pressure and fluid transport due to the passive diffusion across a porous membrane, due to the concentration gradient; and (iii) when the MFC is operating under load, the electro-osmotic drag of water molecules together with the cations that migrate from the anode to the cathode as a consequence of the charge balance (Merino Jimenez et al., 2016). This work aims to analyse the effect of different struvite recovery methods, especially seawater and sea salts, on the power and the catholyte generated from MFCs built from low cost ceramics.

## 2 Materials and methods

### 2.1 MFCs description

**Fig. 1:**
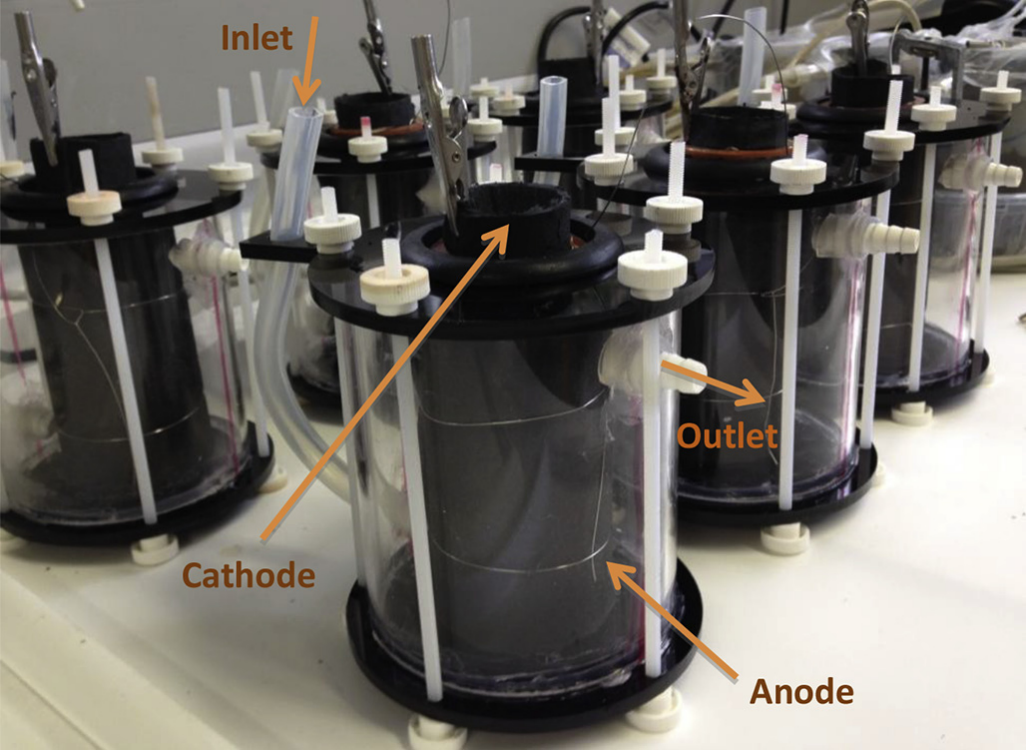
Photo showing the six terracotta cylindrical MFCs.

A total of 6 MFCs were assembled, as shown in Fig. 1, using terracotta ceramic cylinders (Weston Mill Pottery, Newark, Notts, UK) (11 cm height, 42 cm external diameter and 2 mm thickness) as the ion exchange membrane. The anode electrode was prepared from a 90 × 27 cm^2^ piece of carbon veil (30 g/m^2^, PRF Composites, Dorset, UK), which was folded and wrapped around the ceramic cylinder. A stainless steel wire was used to firmly hold the folded electrode to the ceramic material and also acted as the current collector (0.5 mm, Scientific Wire Company). The cathode electrode was prepared by mixing 80 g of activated carbon (GBaldwin&Co, UK) with a solution of 20% polytetrafluoroethylene (PTFE) (60% wt. Sigma-Aldrich). The mixture was then applied onto a piece of carbon veil and hot pressed, resulting in cathode electrodes of 90 cm^2^ that were introduced on the inside of the ceramic cylinders, as previously reported (Gajda et al., 2015). A stainless steel crocodile clip biting on the cathode, acted as the current connector. The MFC structure was introduced in a tubular acrylic container with acrylic lids and was sealed using 5 plastic screws. The anolyte volume of each MFC was 200 ml.

### 2.2 Inoculation

The inoculation was carried out during three consecutive days by inserting a 50:50 mixture of activated sewage sludge (Wessex Water Scientific Laboratory, Saltford, UK) and fresh urine, donated by healthy individuals with no known medical conditions. Two hours after the first inoculation, an external load of 2 kΩ was connected to each MFC. After the third day, a continuous feeding of 100% urine was set up to supply 296 mL per MFC per day giving a retention time of 20.3 h per MFC, using a 16-channel peristaltic pump (205 U, Watson Marlow, Falmouth, UK). The room temperature was 22 ± 2 °C for the duration of the experiment. When the MFCs reached steady state conditions, the external resistance of three MFCs was changed to the optimum value, derived from the polarisation experiments (60 Ω), whereas the other three MFCs were left under open circuit conditions. The three MFCs operating under open circuit conditions were named TS 1, TS 2 and TS 3; while the MFCs working under the optimum load were named TS 4, TS 5 and TS 6, for future references (Fig. 4). The polarisation experiments were performed by connecting a range of external resistances, between the anode and the cathode, from 30 KΩ to 3.74 Ω, for 5 min for each external load, which was sufficient for the MFCs to reach pseudo-steady states. For this purpose, a variable resistor decade box (Centrad Boite A Decades De Resistances DR07 ELC, France) was used. The differences in the maximum power performance from the MFCs using different feedstocks, were statistically analysed using a paired two Samples for Means *t*-test, where p < 0.05.

### 2.3 Feedstock

Five different types of feedstock were tested. Each feedstock was tested for at least one week and fresh urine was used after each type of feedstock, to return to the initial conditions, as a control technique. The urine was collected from a holding tank after 24 h in which the urea was assumed to be completely hydrolysed. After the different test solutions were added to the urine (see below), the mixture was stirred for 30 min and left to sediment for 12 h.

a) **Urine neat with no pre-treatment**.

b) **Urine + MgCl**_**2**_**·6H**_**2**_**O**. According to the IC analysis, urine has an average of 950 ppm of phosphate ions. The best ratio Mg:P reported in the literature is 1:1.2 (Huang et al., 2012, Hutnik et al., 2013a, Hutnik et al., 2013b, Koralewska et al., 2009, Kozik et al., 2011, Matynia et al., 2013), and therefore this is the ratio used during the experiments. A total of 3 g MgCl_2_·6H_2_O per litre of urine was added to fresh urine every day.

c) **Urine neat stirred.**

d) **Urine + artificial sea water (SW)**. Artificial seawater contains a total dry weight of 40.82 g L^−^^1^. In order to maintain the same ratio used in the previous experiments, 0.35 L of artificial seawater was added to each litre of urine used. The seawater was prepared according to previous reports (Anderson, 2008).

e) **Urine + SeaMix** (SMX0015, Peacock Salt, £1.12 Kg^−1^). This commercially available and specially formulated mix is used for simulating seawater when diluted with water (as approved by DEFRA with salinity of 3.5% for the recommended concentration). In order to maintain the same ratio of Mg:P as previously tested, 24.5 g of SeaMix was added to every 2 L of fresh urine.

f) **Urine + deionised water (DIW)**. In order to analyse the separate effect of the urine dilution in water and the addition of sea-salts, 0.35 L of deionised water was added to each litre of urine used as the control condition.

### 2.4 Analysis

To collect the solid samples, after the different feedstocks were left to sediment for 12 h, the supernatant was removed to feed the MFCs, while the remaining slurry was air dried for 48 h until dehydrated and solidified. The remaining solid was weighed and the samples were analysed. A scanning electron microscope (SEM, Philips XL30) was used to capture the images of the struvite crystals. The samples were gold coated at 10 milliamps for 5 min using a PVD Emscope SC500 sputter coating unit. The compositional quality of the crystals can be identified via X-ray diffraction (XRD) by matching the position and intensity of the peaks with the database model for the reference. X-ray diffraction (XRD) patterns were recorded using a Bruker AXS D8 Advance diffractometer with a θ-θ configuration, using Cu Kα radiation (λ = 0.154 nm). The data analysis was performed with Match! Software for phase identification from powder diffraction data, comparing the diffraction pattern of each sample. The phases present were identified by comparison with the software database, which contains the reference patterns, and provided an estimation of the percentage of each compound in the sample. The in-situ synthesised catholyte was collected using a sterile syringe. A Hanna 8424 pH meter (Hanna, UK) and a 470 Jenway conductivity meter (Camlab, UK) were used to measure the pH and conductivity, respectively. Dry weight of precipitated salts was determined by drying 1 mL of catholyte over 48 h and weighing the dry mass. Ion chromatography (IC 930 Compact IC Flex, Metrohm, UK) was used to determine the concentration of anions in the catholyte samples and in the urine. Chemical oxygen demand (COD) analysis was carried out using the potassium dichromate oxidation method (COD HR test vials, Camlab, UK) with an MD 200 photometer (Lovibond, UK) where 0.2 mL samples were taken before and during the MFC treatment and filter-sterilised prior to analysis. A data acquisition/switch unit (Agilent KEYSIGHT, 34972A LXI) was used to monitor the individual MFC voltage levels. Ohm's law was then used to calculate the current and the power output, with the known external resistance that was connected to each cell. The internal resistance (*R*_*INT*_) was calculated from knowing the open circuit voltage (*OCV*) and the current (*I*_*L*_) generated under a given external resistance (*R*_*EXT*_):

(3) $$R_{INT}=\left(\frac{OCV}{I_{L}}\right)-R_{EXT}$$

## 3 Results and discussion

### 3.1 Struvite analysis

**Fig. 2:**
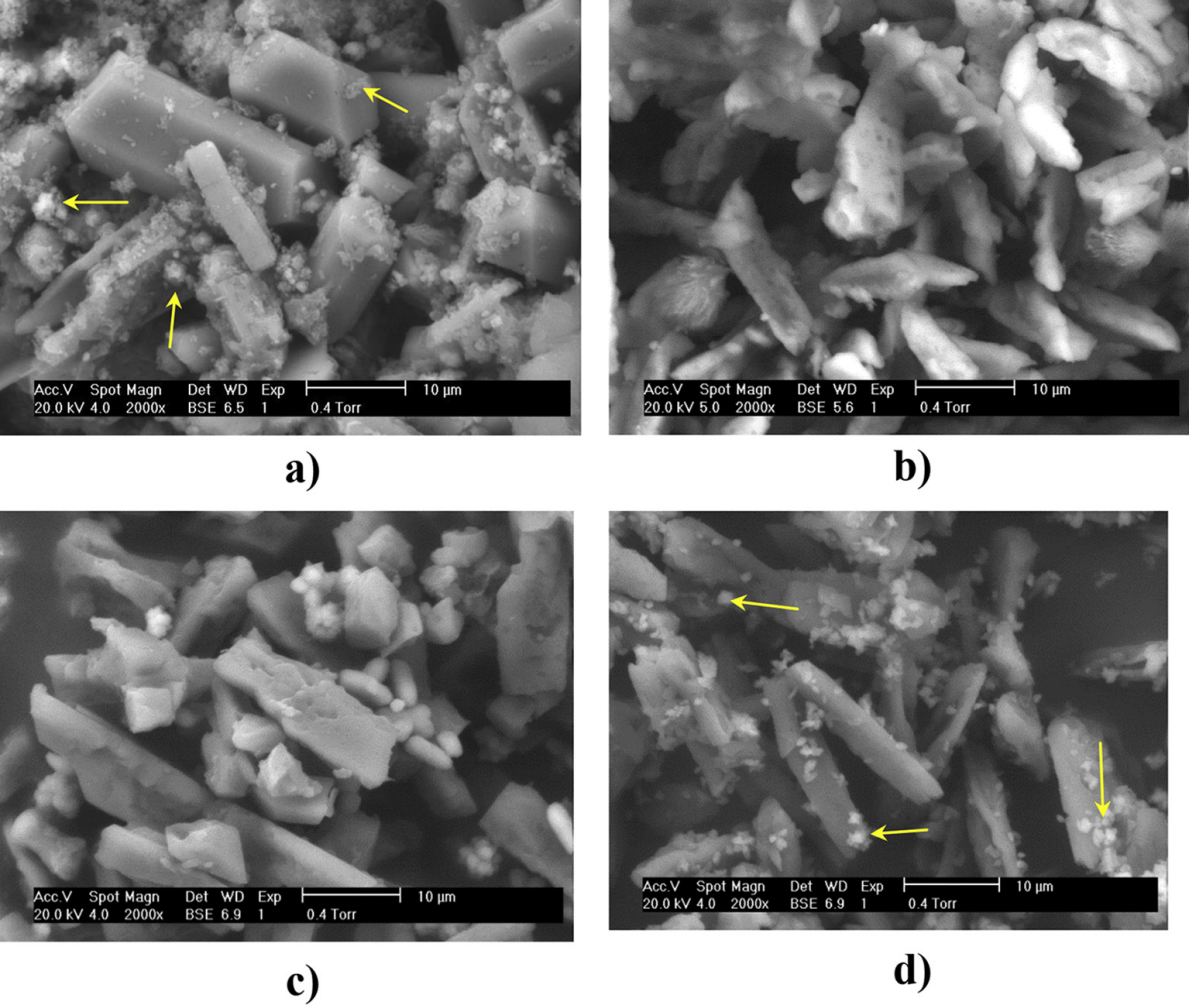
SEM pictures of the struvite crystals obtained from the different precipitation methods: a) urine with no pre-treatment, b) urine + MgCl_2_, c) urine + sea water, d) urine + SeaMix.

Figure 2 shows the SEM pictures of the struvite crystals obtained from the mixtures of urine and the different magnesium sources: (a) urine with no pre-treatment, b) urine + MgCl_2_, c) urine + sea water and d) urine + Sea-Mix. The SEM images only show the results obtained from the feedstocks of interest, since no change (in power) was observed when urine was stirred and in fact power decreased when urine was mixed with deionised water (see Fig. 4). All the SEM images illustrate coarse irregular-shaped crystals with various sizes; the most common morphology observed was the elongated rectangular bar shaped (tabular form) (Zhang et al., 2009). Fig. 2a shows coffin shaped crystals with (001), (101), and (011) faces and different sizes, which is comparable with that obtained by Wierzbicki et al. (1997). Fig. 2b shows crystals with more uniform sizes with an average length of 10 μm, whereas Fig. 2c shows bigger crystals, 20 × 6 μm. X-shaped crystals were also observed in Fig. 2d, which might be due to a higher concentration of peptide in the urine used that led to an interrupted growth from a partial inhibition (Prywer et al., 2012, Wierzbicki et al., 1997). In this figure slightly thinner crystals can be observed, with an average size of 20 × 4 μm.

These results suggest that high growth rates take place in the presence of SeaMix, since X-shaped and elongated crystals were observed in the SEM Figures. Slower crystal growth rates can be expected from urine with no pre-treatment and urine with seawater, since they show tabular crystals (Mclean et al., 1990). Urine with no pre-treatment has a low Mg/PO_4_^3−^ ratio, and therefore a slow growth rate can be expected. By adding seawater to urine, the concentration of Mg increases whereas the concentration of PO_4_^3−^ decreases due to the urine dilution, leading to a slow growth rate. Apart from the struvite crystals, there might also be solids precipitation from amorphous calcium phosphate, halite or brucite (Capdeviellea et al., 2013). The presence of the typical clusters of calcium phosphate, which are usually found in raw stored urine, can also be observed (marked with a yellow arrow), especially in Fig. 2a.

**Fig. 3:**
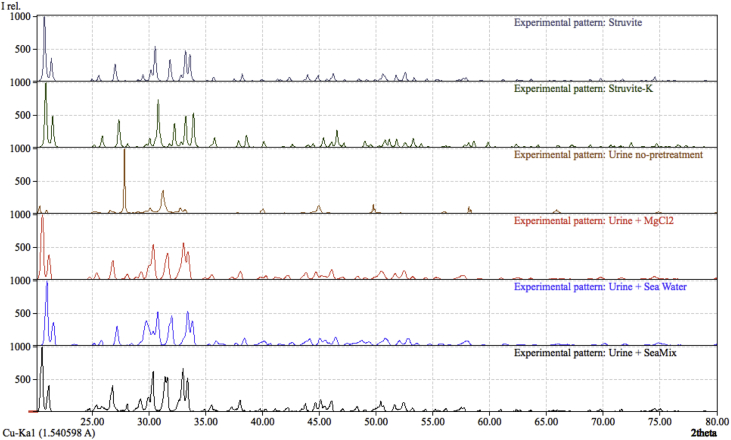
XRD of the struvite obtained from different feedstocks in comparison with the Struvite (reference: 96-900-7675) and Struvite-K (reference: 96-901-0848) patterns.

The XRD analysis of the struvite crystals obtained from urine mixed with different magnesium sources is shown in Fig. 3. The XRD pattern position and intensity of the peaks generated from the struvite crystals matched the reference values of pure struvite in different percentages for the magnesium sources added. The urine with no pre-treatment produced crystals but only 21.05% of the solid matter was struvite. According to the XRD analysis, when urine was mixed with MgCl_2_, 80.84% of the sample was struvite (52.07% MKP and 28.77% MAP). A slightly lower percentage of struvite 72.7% was obtained from the samples collected from urine mixed with artificial sea water. When the mixture of urine and SeaMix was used, up to 94% of the collected solid was struvite, including MKP, MAP and arsenstruvite, according to the XRD pattern. A higher percentage of MKP was obtained in this case, due to a higher concentration of potassium compared to ammonium. These results suggest that among the magnesium sources used in this work, the most efficient in terms of struvite precipitation was the SeaMix, obtaining the highest percentage of struvite crystals.

### 3.2 Effect of the addition of Mg sources to urine in the MFCs power production and catholyte accumulation

**Fig. 4:**
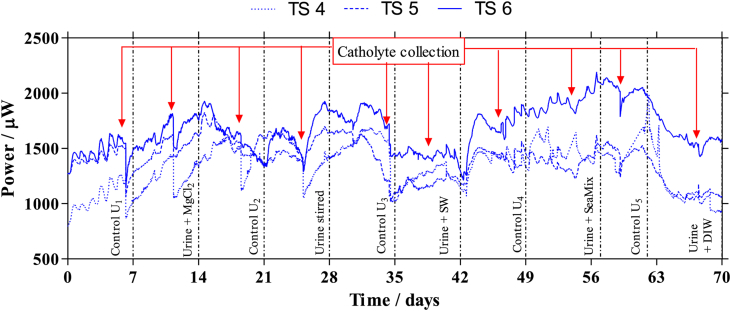
Temporal power performance from the loaded MFCs, under the different feeding conditions.

Fig. 4 shows the performance of the loaded MFCs when using different feedstocks. Urine was used as the feedstock during the first 7 days shown in the graph, producing an average power output of 1300 μW per single MFC. On day 6, the catholyte was completely drained causing a power drop of over 400 μW, which was then recovered in approximately 24 h when new catholyte was generated. The next 7 days, urine + MgCl_2_ resulted in a continuous increase in power performance. The average power reached a maximum of 1600 μW, when the catholyte was again drained causing a decrease in performance that was again recovered after approximately one day. From day 14 the feedstock was changed to urine (control U_2_). At day 15, the performance reached 1865 μW, probably due to residual MgCl_2_ in the anolyte, since the retention time was 20.3 h. After a complete replacement of the anolyte in the MFCs, the performance continuously decreased to 1450 μW. As shown in Fig. 4, no significant effect was observed during the subsequent three weeks, only in the presence of urine + sea water the performance decreased to an average of 1250 μW, most likely due to the urine being diluted, since performance rose again to 1500 μW after feeding the MFCs with urine (control U_4_). However, it was when the mixture of urine and SeaMix was used, that the system reached its maximum power output for the whole experiment, with an average of 1800 μW, decreasing slightly when only fresh urine was used again (control U_5_) but more rapidly with diluted urine (urine + DIW), to 1166 μW.

Table 1 shows a comparison of the properties of the different feedstocks (pH and conductivity), the amount of precipitate obtained from each mixture and the percentage of struvite recovered, together with the maximum power generated from the polarisation experiment per individual MFC, the internal resistance and the properties of the catholyte collected (amount, pH and conductivity). As can be seen from the table, no significant effect in power, internal resistance or electrolyte conductivity was observed by the addition of MgCl_2_ in comparison with the control U_1_. This suggests that although the addition of MgCl_2_ may be beneficial for struvite precipitation, it does not affect the power production from the MFCs. However, the addition of seawater to the urine caused a significant difference in the power performance (T-test, p = 0.01), decreasing the maximum power performance from the polarisation from 1769 to 1394 μW. This was probably due to the urine being diluted and not because of a negative effect of seawater to the system, since the addition of SeaMix to urine led to an 11.3% increase in power performance, compared to urine alone (T-test, p = 0.03). This increase may be due to an increase in the conductivity of the anolyte, which decreased the internal resistance of the system. Moreover, the addition of SeaMix led to the highest amount of struvite precipitated, 5.1 g, at the highest concentration of 94% of the solids. These results suggest that the addition of sea salt is beneficial in terms of struvite recovery and power performance, but the addition of water dilutes urine and decreases the power generated by at least 20%. The dilution of urine with deionised water produced a more pronounced decrease in power of over 35% (T-test, p = 0.02), since the conductivity decreased to 21.2%.

The COD removal slightly increased in the presence of SeaMix, increasing from 16.2% when urine + MgCl_2_ was used, to 18.1% when Urine + SeaMix was used. The increase in power generated and the increment in COD reduction would also increase the coulombic efficiency of the system. Tang et al. (2014) also showed an increase in the coulombic efficiency from 9.7 to 26.6% when using a phosphate free anolyte.

Table 1 also shows the amount of catholyte collected, its pH and conductivity, when the different feedstocks were tested in the terracotta MFCs, under open circuit conditions and under a 60 Ω load. The amount of catholyte collected from the MFCs under OCV was considerably lower than that obtained under load, which is in agreement with previous work (Gajda et al., 2014). Gajda et al. (2014) suggested that the catholyte generation in ceramic MFCs under load is due to the combination of active and passive diffusion, including electro-osmotic drag. In this study, the power output of the MFCs varied depending on the feedstock used, and therefore, the amount of catholyte collected and its properties were also expected to change. At higher power, a higher concentration of OH^−^ is dragged through the membrane and more H_2_O_2_ molecules are produced in the cathode, leading to a more alkaline catholyte. However, the catholyte produced is not only dependent on the power generation, but also on the anolyte properties (Pivovar, 2006). If the conductivity or the pH of the anolyte drastically changes, the amount of catholyte produced, its conductivity and pH will also change. As can be seen in Table 1, less catholyte with higher conductivity and pH was generated when the anolyte also had a higher conductivity and a high concentration of salts. The urine dilution with deionised water, led to a less conductive anolyte solution, which produced a larger amount of catholyte with lower conductivity and pH.

This can be explained by the electro-osmotic drag coefficient, which gives the number of water molecules that accompanies each particular ion through the ceramic membrane (Pivovar, 2006). The electro-osmotic drag coefficient values for a specific ion might vary with the type of membrane and its porosity. However, evidence of differences in the electro-osmotic drag coefficient of particulars ion, depending on their identity, has also been reported. The drag coefficient of K^+^ has been reported to be 11 (Okada et al. 1992), whereas for Na^+^ was 10.9 and for Cl^−^ was two-fold lower, 4.8 (Liam et al., 2014). The precipitation of K-struvite from the addition of sea salts to urine suggests a decrease in the number of K^+^ ions in the anolyte and catholyte, which decreased the number of water molecules dragged from anolyte to catholyte. On the contrary, by increasing the concentration of Cl^−^ in the anolyte, by adding MgCl_2_ to the urine, it would be expected that the amount of catholyte accumulated would increase, since more Cl^−^ would pass through the membrane. However, Liam et al. (2014) reported a decrease of the Cl^−^ drag coefficient from 10.9 to 4.8 when increasing the concentration of Cl^−^ from 1.5 to 6 M. This may explain the decrease in the amount of catholyte collected, in the present study, when the concentration of Cl^−^ was higher. This increase in the electro-osmotic drag coefficient with a lower concentration of ions may be due to the increase of the water content in the membrane and the consequent increase of the effective size of water domains, along with the ion conducting phase, in the nanostructure of the membrane (Liam et al., 2014).

**Fig. 5:**
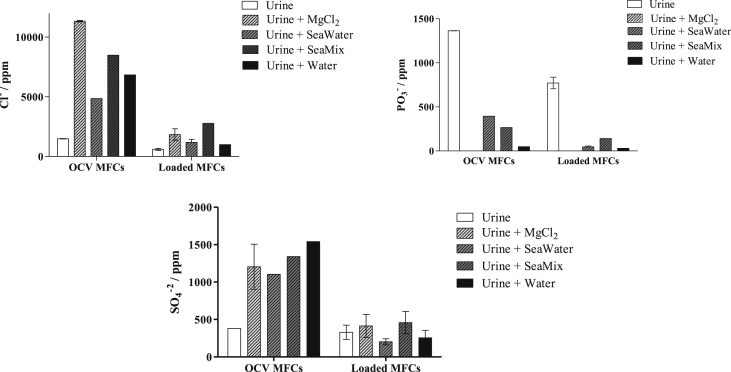
Chloride, phosphate and sulphate anion concentrations of the catholyte collected from the MFCs when fed with different inlets.

The concentrations of Cl^−^, PO_3_^−^ and SO_4_^−^ in the catholyte collected for the different feedstocks are shown in Fig. 5. A lower concentration of anions was observed in the catholyte from MFCs under load, in comparison with the catholyte from MFCs under open circuit. Under load, positive charged molecules travel from anode to cathode as a consequence of electro-osmosis, which is a charged-transfer based process to balance the negative charge of the electrons travelling from anode to cathode. As expected, in the presence of any Mg source, the concentration of phosphate ions decreased dramatically since it precipitated as struvite. On the contrary, the concentration of sulphate ions in the catholyte collected from the loaded MFC was not affected by the addition of any magnesium source.

As the results show, the anolyte composition has a great effect on the catholyte generated. This provides an excellent opportunity for tuning the system in terms of varying the concentration of the different components in the anolyte, in order to obtain a catholyte with the desired composition. The investigation about catholyte production from ceramic MFCs is at an early stage and the qualitative and quantitative effect of the different parameters that affect the catholyte generation and its quality needs to be addressed. A user-defined catholyte, can then be ‘designed’, which can be used for practical applications, could be synthesised in-situ.

## 4 Conclusions

This work presents a low cost system capable of producing electricity, generating catholyte, treating urine and recovering struvite. Low cost MFCs were prepared using terracotta clay as the membrane material, cathode electrodes based on activated carbon and carbon veil as the anode material. The addition of SeaMix, a commercially available sea salt preparation, showed an increase in the struvite recovery reaching 94% and increasing the maximum power performance of the MFCs by more than 10%. The catholyte generation was also affected by the addition of the SeaMix, increasing the pH and conductivity. The production cost of the struvite precipitated with SeaMix was £0.027 g^-1^, which is considerably lower than that produced from urine mixed with MgCl_2_, £0.21 g^-1^. Moreover, the commercial SeaMix could be potentially substituted by sea salts from natural sources, by evaporating real sea water. These results confirm the possibility of a MFC capable of producing electricity, catholyte and struvite at no extra cost than the current manufacturing process. The combination of urine and seawater may also open up other opportunities, for reducing salinity in waste-streams, and this can form part of future work.

## Figures and Tables

**Table 1:** Properties of the different feedstocks, precipitate obtained from the mixture, struvite recovered; together with the maximum power generated per individual MFC, the internal resistance and the properties of the catholyte collected.

Feedstock	Amount of solids collected/g	Struvite present in the solids/%	Maximum Power according to polarisation/μW	*R*_*INT*_*(Ω)*	Feedstock pH	Feedstock Conductivity/mS s^−1^	Catholyte collected/ml day^−1^		Catholyte pH		Catholyte Conductivity/mS s^−1^	
							OCV	Loaded	OCV	Loaded	OCV	Loaded
Control U_1_	2	21	1764 ± 78	40.73	9.2	23.2	1.19	3.6	9.295	10.41	21.3	20.73
Urine + MgCl_2_	4.9	80	1756 ± 115	40.37	9.2	28.6	0.52	3.38	9.175	10.36	21.8	22.1
Control U_2_			1966 ± 156	40.75	9.28	31.8	0.71	3.04	9.15	10.45	23.5	24.5
Urine Stirred			1799 ± 99	40.7	9	30.5	0.55	2.8	9.18	10.15	23.6	25
Control U_3_			1769 ± 167	40.75	9.23	31	0.53	2.4	9.20	10.12	23.6	26
Urine + SeaWater	4.75	73	1394 ± 99	48.35	9.25	43.6	0.14	3.09	8.89	10.44	23.1	26.36
Control U_4_			1830 ± 237	40.72	9.25	28.7	0.357	2.5	9.05	10.65	26.2	27.86
Urine + SeaMix	5.1	94	2055 ± 268	34.14	9.25	43.6	0.41	1.57	9.42	10.62		36.3
Control U_5_			2089 ± 300	34.14	9.22	30	0.64	1.35	8.95	10.21	31.9	37.46
Urine + DI water			1316 ± 150	48.35	9	21.2	0.328	2.12	8.88	9	27.5	31.3
